# Marital control, domestic violence justification, and mental health: a study of married women in Bangladesh

**DOI:** 10.1186/s12888-025-07133-3

**Published:** 2025-09-30

**Authors:** Mortuja Mahamud Tohan, Sayeeda Zaman, Bristi Rani Saha, Nushrath Jahan Juhi, Shaiya Binte Mahbub, Md. Hasan Howlader

**Affiliations:** 1 School of General Education (GenEd), Brac University, Dhaka, Bangladesh; 2 Development Studies Discipline, Social Science School, Khulna University, Khulna, 9208 Bangladesh

**Keywords:** Depression, Anxiety, Domestic violence, Decision making, Bangladesh

## Abstract

Marital control and culturally accepted justifications of domestic violence are pervasive forms of gendered power imbalances that undermine women’s autonomy and significantly contribute to adverse mental health outcomes. These factors are critical yet often overlooked in patriarchal settings like Bangladesh, where the interpersonal dynamics within marital households pose a substantial health burden, with long-lasting effects on the well-being of women. Despite this, national-scale studies remain scarce. This study examines how attitudes toward domestic violence and socioeconomic contexts influence anxiety and depression among married women in Bangladesh, leveraging data from the 2022 Bangladesh Demographic and Health Survey (BDHS). Anxiety and depression were assessed using the Generalized Anxiety Disorder (GAD-7) and Patient Health Questionnaire (PHQ-9) scales. Descriptive statistics, one-way ANOVA, binary logistic regression, and stepwise multiple regression were employed to identify predictors. Results indicate that 26.9% and 29.2% of married women experienced mild-to-severe anxiety and depression, respectively, with 4.5% reporting moderate-to-severe anxiety and 1.3% moderate-to-severe depression. Justified domestic violence was significantly associated with higher odds of moderate to severe anxiety and depression. Women who believed wife-beating was acceptable in situations such as going out without informing the husband (*p* < 0.001), neglecting children (*p* < 0.001), arguing (*p* < 0.001), refusing sex (*p* < 0.001), or burning food were more likely to suffer from anxiety and depression. Stepwise regression identified terminated pregnancy as the strongest predictor of both anxiety (R^2^ = 0.071, *p* < 0.001) and depression (R^2^ = 0.069, *p* < 0.001). Socioeconomic factors, such as education and continuing education after marriage, also significantly influenced outcomes. The findings underscore the high prevalence of mental health issues among Bangladeshi married women, shaped significantly by socioeconomic conditions and normalization of gender-based violence. These findings highlight the urgent need for culturally sensitive interventions addressing familial pressures and gender-based violence. Future research should employ longitudinal designs to explore causal pathways and incorporate additional determinants, such as social support and coping mechanisms, to inform comprehensive mental health policies in low-resource settings.

## Introduction

Marital control and domestic violence continue to be widespread issues around the world, especially in areas where patriarchal systems and traditional gender norms dictate societal expectations. Marital control encompasses a pattern of coercive behaviors exerted by one partner in a marital or intimate relationship, aimed at dominating, restricting, or regulating the autonomy and decision-making of the other partner [1]. These behaviors are often rooted in power imbalances and are associated with broader social and relational dynamics that justify or normalize domestic violence [1]. Justification of Domestic violence refers to the belief or attitude that physical, emotional, or sexual abuse by a spouse or partner is acceptable or justifiable under certain conditions [2]. Such justification perpetuates violence against women, which encompasses physical, sexual, or psychological harm that causes suffering or restricts women’s individual rights and societal freedom [3]. It is estimated that 50% of women in low-income nations believe that wife battering or physical and/or sexual violence by male partner is acceptable [4]. Bangladesh, a culturally conservative nation with strict gender roles, represents one such context, where women's freedom is often restricted by societal norms [5, 6].

Domestic violence has profound implications for women’s psychological well-being, self-esteem, and overall health [7]. Psychological well-being is described as a sense of contentment, fulfillment, and enjoyment in many aspects of life, including marital relationships [8]. According to the World Health Organization (WHO), 37% of women globally have experienced intimate partner violence (IPV) at some point in their lives. IPV not only leads to physical harm but also has lasting effects on women’s mental health, contributing to conditions such as depression, anxiety, and stress, which are exacerbated by experiences of abuse [9, 10].

Prior research demonstrated that marital relationships characterized by restricted personal autonomy in decision-making, diminished independence, and unbalanced power dynamics negatively impact women's mental well-being. This can manifest in the development of psychological disorders, such as clinical depression—including suicidal thoughts and suicide attempts—as well as anxiety stemming from experiences of abuse within the marriage [11, 12].

Studies indicate that women who have experienced IPVs are significantly more likely to develop anxiety disorders [13, 14]. Anxiety disorders, in turn, can lead to substantial functional impairments, reduced quality of life, and an increased risk of developing comorbid mental health conditions like depression [15, 16]. Women with anxiety often experience persistent sadness, a lack of interest in once-pleasurable activities, and feelings of hopelessness, guilt, and worthlessness, with some suffering from symptoms like insomnia, loss of appetite, and unintended weight loss [17, 18].

Depression is one of the most prevalent mental health consequences for female survivors of IPVs, with prevalence rates ranging from 21% to as high as 83%, depending on the geographic region examined [19]. In some areas, violence against women is not only widespread but also culturally and socially acceptable. For example, in certain regions of the world, it is viewed as justifiable for men to beat their wives, often due to entrenched beliefs about gender roles and marital control [20, 21]. This cultural acceptance creates a vicious cycle of abuse, where women are conditioned to either tolerate or expect mistreatment from their husbands. Marital control, as a form of personal abuse, is inherently tied to broader societal attitudes towards gender inequality, which are often reinforced within the family and community [22, 23].

Research from countries like India and regions such as Sub-Saharan Africa shows that cultural norms often justify intimate partner violence, highlighting the global normalization of abuse across patriarchal societies [24]. Existing research also highlights troubling trends in Bangladesh, where a notable 28% of women surveyed believe it is acceptable to physically beat one's wife [25]. When domestic violence is seen as a normal or justifiable aspect of marriage within a society, it serves to significantly undermine the agency and self-worth of victimized women. This pervasive normalization often leads to profound psychological distress, as survivors may internalize a sense of blame for the abuse inflicted upon them. Such internalization can foster debilitating feelings of guilt, helplessness, and despair. The victim's own self-perception becomes indelibly shaped by the abuse, further trapping them in cycles of violence and poor mental health [23].

While several studies have indeed focused on the prevalence and impacts of domestic violence within South Asia more broadly [26, 27] these investigations often fail to adequately account for the critical cultural factors that influence how such violence is justified or normalized within the institution of marriage. Importantly, the ways in which these justifications may contribute to the deterioration of women's mental health have been largely overlooked [28]. Although domestic violence has been widely studied in South Asia, limited research has addressed how cultural justifications of abuse within marriage affect women's mental health, particularly in the context of Bangladesh. This present study aims to address this significant gap in the scholarly understanding. By closely examining the intricate relationship between marital control, the justification of violence, and mental health outcomes among married women in Bangladesh, it seeks to provide crucial insights into how prevailing cultural norms surrounding gender roles and the acceptability of domestic abuse ultimately impact the psychological well-being of this vulnerable population.

### Theoretical framework

This study employs the Stress Process Theory to elucidate how marital control, justification of domestic violence, and contextual factors collectively shape mental health outcomes (anxiety and depression) among women in Bangladesh, which has been used to explain mental health of women in other previous studies [29] At its core, the framework identifies marital control, manifested through restricted decision-making autonomy (healthcare, contraception, mobility), as a chronic primary stressor. Prolonged exposure to such control erodes women’s agency, fostering persistent psychological strain, helplessness, and emotional exhaustion [30] Concurrently, justification of domestic violence (believing husbands are entitled to beat wives for transgressions like arguing or neglecting chores) acts as a normative stressor, normalizing abuse and internalizing self-blame. This internalization creates cognitive dissonance, where women may rationalize their victimization, deepening feelings of guilt and worthlessness [31].

Secondary stressors, such as reproductive coercion (e.g., pressure to terminate pregnancies or conceive) and social restrictions (e.g., continuing work and education after marriage), compound these primary stressors. For instance, societal norms isolating women who defy marital control amplify isolation, exacerbating distress [32]. The theory further accounts for resources and moderators that buffer or intensify stress impacts [33]. Higher education and wealth enhance coping capacity by empowering women to challenge oppressive norms or seek support. Employment and digital access (e.g., internet use) act as protective resources, fostering economic independence and social connectivity, which counterbalance feelings of entrapment.

Covariates contextualize these dynamics. Early age at first sex or childbirth reflects limited autonomy, heightening vulnerability to stress. Cultural and religious norms (e.g., patriarchal interpretations of marital roles) reinforce DV justification as a socially sanctioned stressor. Conversely, decision-making autonomy (e.g., independent/joint choices about visiting relatives) serves as a protective factor, bolstering self-esteem and resilience. Collectively, these elements create a stress pathway: marital control and domestic violence justification generate chronic stress, which, when unmitigated by resources, overwhelms coping mechanisms, leading to anxiety and depression.

## Method and measurements

### Data source and sampling

This study utilized secondary data from the 2022 Bangladesh Demographic and Health Survey (BDHS), which employed a two-stage stratified sampling approach. In the first stage, 438 rural and 237 urban enumeration units were selected using a probability proportional to size sampling technique, followed by a complete listing of households within each selected unit. In the second stage, systematic sampling was applied to select 30 households from each enumeration unit. The survey ultimately interviewed 30,078 households from 675 clusters (237 urban and 438 rural) across eight divisions of Bangladesh. Further details on the sampling procedures are available elsewhere [34]. For this study, a total of 8,593 women of reproductive age were included after excluding those with missing height or weight data, as well as women who were pregnant or had given birth within two months prior to data collection.

### Outcome variables

The 2022 BDHS mental health module incorporated two widely used screening tools for mental disorders: the Generalized Anxiety Disorder (GAD-7) and the Patient Health Questionnaire (PHQ-9), which assess anxiety and depression, respectively. Both instruments have been previously validated in Bangladesh and demonstrated strong reliability [35]. These scales evaluate symptoms experienced within the two weeks prior to the survey. The GAD-7 categorizes anxiety severity, with scores of 0–5 indicating mild anxiety, 6–14 indicating moderate anxiety, and 15–21 indicating severe anxiety [36]. Similarly, the PHQ-9 classifies depression severity, where scores of 0–4 represent minimal or no symptoms, 5–9 indicate mild symptoms, 10–14 correspond to moderate symptoms, 15–19 signify moderately severe symptoms, and 20–27 reflect severe symptoms [37]. Both instruments utilize a Likert scale to measure symptom severity, assigning numerical values to responses:"not at all"(never),"several days"(rarely),"more than half the days"(often), and"nearly every day"(always), scored as 1, 2, and 3. The final score is obtained by summing the individual item scores. Additionally, in the 2022 BDHS, the validated Water Glass Pictorial Scale was used to visually represent symptom severity. An empty glass signified"never,"a quarter-full glass represented"rarely,"a half-full glass denoted"often,"and a three-fourths full glass indicated"always."This pictorial representation helped respondents better express their experiences and provided contextual understanding of symptom severity. However, no diagnostic testing was conducted. The reliability of the scales in this sample was reflected in Cronbach’s alpha values of 0.831 for PHQ-9 and 0.884 for GAD-7.

### Predictor variables

To identify potential predictor variables linked to the outcome measures, an extensive review of the literature was carried out. Drawing from the existing dataset and prior research, a variety of factors were chosen for analysis, encompassing biological, socioeconomic, behavioral, and domestic violence-related dimensions (Table 1). Socioeconomic factors included age, geographic region, level of education, wealth status, type of residence (urban or rural), marital status, religious affiliation, and employment status. Biological and behavioral variables consisted of age at first sexual intercourse, history of pregnancy terminations, contraceptive use, smartphone ownership, and internet usage.

**Table 1:** Construct of independent variables

Code	Independent Variables	Categories
V1	Age	15–24 Years, 25–34 Years, 35–49 Years
V2	Status of Marriage	Currently married, Separated/Deserted/Divorced, Widowed
V3	Education	No education, Primary, Secondary/Higher
V4	Household wealth status	Poor, Middle, Rich
V5	Religion	Islam, Hinduism/other religion
V6	Place of Residence	Urban, Rural
V7	Respondent currently employed	No, Yes
V8	Age of having first sex	< 18, ≥ 18
V9	Age of first birth	< 18, ≥ 18
V10	Own mobile phone	No, Yes
V11	Own Smart phone	No, Yes
V12	Ever Used Internet	No, Yes
V13	Ever had a terminated pregnancy	No, Yes
V14	Ever used anything to delay or avoid getting pregnant	No, Yes
V15	Decision on contraceptive use	Respondent alone/with partner, Husband/partner alone, Other Interfere
V16	Decision on health care visit	Respondent alone/with partner, Husband/partner alone, Other Interfere
V17	Decision on visiting relative house	Respondent alone/with partner, Husband/partner alone, Other Interfere
V18	Husband Education	No education, Primary, Secondary/Higher
V20	Husband or family member pressured respondent to become pregnant	No, Yes
V21	Continued study after marriage	No, Yes
V22	Continued work after marriage	No, Yes
V23	Beating justified if wife goes out without telling husband	No, Yes
V24	Beating justified if wife neglects the children	No, Yes
V25	Beating justified if wife argues with husband	No, Yes
V26	Beating justified if wife refuses to have sex with husband	No, Yes
V27	Beating justified if wife burns the food	No, Yes

To capture marital control and women’s decision-making power, both recognized in the literature as critical indicators of agency and exposure to controlling behavior, we used three specific BDHS questions: “Who usually makes decisions about contraceptive use?”, “Who usually makes decisions about your health care?”, and “Who usually makes decisions about visiting relatives?” Responses were recategorized into three groups: (1) decisions made by the respondent alone or jointly with her partner (reflecting autonomy), (2) decisions made by the husband/partner alone (indicating less autonomy), and (3) decisions made by others, suggesting external interference.

In addition, to assess normative acceptance of domestic violence, we included five attitude-based questions from the BDHS Women’s Questionnaire. Women were asked whether a husband is justified in hitting or beating his wife under specific circumstances. The scenarios included: if the wife goes out without telling him, neglects the children, argues with him, refuses to have sex with him, or burns the food. Each response was recorded as a binary variable (Yes = 1, No = 0). These items (coded in BDHS as d105a to d105e) are widely used in international research as indicators of gender norms and societal tolerance of domestic violence.

### Statistical analysis

This study employed both descriptive and inferential statistical methods for data analysis. Descriptive statistics were used to summarize the background characteristics of the respondents, providing a comprehensive overview of their demographic and socioeconomic profiles.

For inferential analysis, the primary focus was on identifying potential predictor variables associated with moderate to severe anxiety and depression among married women in Bangladesh. To achieve this, unadjusted binary logistic regression analyses were conducted. These models assessed the bivariate relationships between each independent variable and the dichotomized outcome variables. Anxiety measured using the Generalized Anxiety Disorder (GAD-7) scale, categorized into minimal/no anxiety (0–4) vs. moderate to severe anxiety (5–21), and Depression measured using the Patient Health Questionnaire (PHQ-9), categorized into minimal/no depression (0–4) vs. moderate to severe depression (5–27).

All independent variables were included in the unadjusted models to explore their individual associations with the outcomes. No adjusted (multivariate) logistic regression models were developed. This decision was made to maintain the exploratory nature of the study and to avoid potential issues related to multicollinearity and overfitting, especially given the sample size and distribution of responses. As such, Table 2 presents only unadjusted odds ratios (ORs), offering insight into the crude associations without controlling for covariates.

**Table 2:** Binary logistic regression of independent variables and GAD and PHQ scores

	GAD Score		PHQ Score	
Variables	UOR (95% CI)	Sig	UOR (95% CI)	95% CI)
Age				
15–24 Years	1		1	
25–34 Years	1.12 (0.85–1.48)	0.429	0.89 (0.70–1.15)	0.372
35–49 Years	1.17(0.89–1.60)	0.257	0.94 (0.73–1.20)	0.599
Status of Marriage				
Currently married	1		1	
Separated/Deserted/Divorced	1.561 (1.21–1.95)	< 0.001	1.86 (1.54–2.24)	< 0.001
Widowed	2.225 (1.54–2.67)	< 0.001	1.95 (1.64–2.31)	< 0.001
Education				
No education	1.84 (1.69–2.02)	< 0.001	1.44 (1.32–1.57)	< 0.001
Primary	1.36 (1.19–1.56)	< 0.001	1.23 (1.08–1.41)	0.002
Secondary/Higher	1		1	
Household wealth status				
Poor	1.35 (1.18–1.54)	< 0.001	1.10 (0.97–1.26)	0.005
Middle	1.17 (1.02–1.35)	0.027	1.15 (1.00–1.31)	0.046
Rich	1		1	
Religion				
Islam	1.31 (1.09–1.58)	0.004	1.15 (0.97–1.36)	0.121
Hinduism/other religion	1			
Respondent currently employed				
No	1		1	
Yes	1.14 (1.07–1.22)	< 0.001	1.14 (1.03–1.27)	0.014
Age of having first sex				
< 18	1.23 (1.15–1.31)	< 0.001	1.14 (1.07–1.22)	< 0.001
≥ 18	1		1	
Age of first birth				
< 18	1.18 (1.10–1.26)	< 0.001	1.14 (1.07–1.21)	< 0.001
≥ 18	1		1	
Own Smart phone				
No	0.95 (0.82–1.10)	0.462	0.91 (0.79–1.05)	0.192
Yes	1		1	
Ever Used Internet				
No	1.30 (1.119–1.507)	0.001	1.22 (1.06–1.41)	0.006
Yes	1		1	
Ever had a terminated pregnancy				
No	1		1	
Yes	0.72 (0.64–0.81)	< 0.001	1.31 (1.22–1.41)	< 0.001
Ever used anything to delay or avoid getting pregnant				
No	1.05 (0.89–1.24)	0.536	0.90 (0.77–1.04)	0.159
Yes	1		1	
Decision on health care visit				
Respondent alone/with partner	1.15 (0.71–1.84)	0.579	0.84 (0.56–1.27)	0.408
Husband/partner alone	1.11 (0.68–1.80)	0.686	0.87 (0.57–1.32)	0.512
Other Interfere	1		1	
Decision on visiting relative house				
Respondent alone/with partner	2.03 (1.44–2.86)	< 0.001	1.83 (1.35–2.49)	< 0.001
Husband/partner alone	1.89 (1.32–2.71)	< 0.001	1.87 (1.35–2.57)	< 0.001
Other Interfere	1			
Husband Education				
No education	1.12 (0.94–1.33)	0.222	0.95 (0.80–1.13)	0.565
Primary	1.06 (0.93–1.21)	0.380	0.95 (0.83–1.07)	0.383
Secondary/Higher	1		1	
Continue work after marriage				
No	1		1	
Yes	2.00 (1.52–2.63)	< 0.001	1.82 (1.39–2.39)	< 0.001
Continued study after marriage				
No	1.04 (0.90–1.20)	0.594	1.14 (1.00–1.31)	0.051
Yes	1		1	
Beating justified if wife goes out without telling husband				
No	1		1	
Yes	1.59 (1.41–1.78)	< 0.001	1.46 (1.30–1.64)	< 0.001
Beating justified if wife neglects the children				
No	1		1	
Yes	1.45 (1.15–1.83)	0.002	1.33 (1.19–1.49)	< 0.001
No	1		1	
Yes	1.40 (1.26–1.55)	< 0.001	1.25 (1.12–1.38)	< 0.001
Beating justified if wife refuses to have sex with husband				
No	1		1	
Yes	1.71 (1.45–2.00)	< 0.001	1.49 (1.27–1.74)	< 0.001
Beating justified if wife burns the food				
No	1		1	
Yes	1.79 (1.44–2.23)	< 0.001	1.44 (1.15–1.80)	0.001

-UOR = Unadjusted odd ratio

-Dependent variables

1: Depression = 0: Minimal Depression (reference category); 1: Mild to severe Depression

2: Anxiety = 0: Minimal Anxiety (reference category); 1: Mild to severe Anxiety

In addition, stepwise multiple regression analysis was conducted as an exploratory method to assess the collective influence of multiple predictor variables on the continuous outcome scores of anxiety and depression. It was selected due to its utility in exploratory research for variable selection when theoretical guidance is limited.

To address potential concerns about overfitting in the stepwise multiple regression model, a tenfold cross-validation procedure was performed. The dataset was randomly divided into 10 subsets of approximately equal size. In each iteration, the model was trained on 9 folds and validated on the remaining fold. This process was repeated 10 times.

For the anxiety model (GAD-7 score as a continuous outcome), the average cross-validated Root Mean Squared Error (RMSE) was 3.82, and the average Mean Absolute Error (MAE) was 2.91, indicating a moderate level of predictive accuracy. The cross-validated R-squared was 0.17, suggesting that approximately 17% of the variance in anxiety scores could be explained by the predictor variables retained in the model. Similarly, for the depression model (PHQ-9 score), the average RMSE was 4.45, with an MAE of 3.42 and an R-squared value of 0.21, demonstrating a slightly better fit than the anxiety model. All statistical analyses were performed using STATA MP 16 and SPSS version 26 software packages.

## Result

### Background characteristics

Table 3 highlights key demographic characteristics of the study population (30,078). The majority of participants (61.8%) fell within the 35–49 years age group. A significant proportion were married (94.9%) and was living in rural areas (64.9%). More than half of the women (60%) achieved at least a secondary-level education, with 42.3% coming from affluent households. Most of their husbands were aged between 31 and 50 years (60.4%), and with more than half (50.9%) having attained secondary education or higher. Notably, 81.0% of women reported discontinuing their studies after marriage, and 51.0% did not continue working post-marriage. About one-third (30.9%) of the women were employed. A large majority (89.6%) identified as Muslim. Regarding mobile access, 69.5% of the respondents owned a mobile phone, with 45.7% owning a smartphone. However, 70.9% had never used the internet.

**Table 3:** Frequency distribution of the variables of the included in the study

Variables	Frequency	Percentage (%)
Age		
15–24 Years	1049	3.5
25–34 Years	10,427	34.7
35–49 Years	18,602	61.8
Status of Marriage		
Currently married	28,537	94.9
Separated/Deserted/Divorced	675	2.2
Widowed	866	2.9
Education		
No education	4167	13.9
Primary	7857	26.1
Secondary/Higher	18,054	60.0
Household wealth status		
Poor	11,414	37.9
Middle	5926	19.7
Rich	12,738	42.3
Religion		
Islam	26,958	89.6
Hinduism/other religion	3120	10.4
Place of Residence		
Urban	10,571	35.1
Rural	19,507	64.9
Respondent currently employed		
No	13,812	69.1
Yes	6175	30.9
Age of having first sex		
< 18	13,112	65.6
≥ 18	6875	34.4
Age of first birth		
< 18	10,537	39.1
≥ 18	16,396	60.9
Own mobile phone		
No	9186	30.5
Yes	20,892	69.5
Own Smart phone		
No	11,342	54.3
Yes	9550	45.7
Ever Used Internet		
No	14,168	70.9
Yes	5819	29.1
Ever had a terminated pregnancy		
No	23,341	77.6
Yes	6737	22.4
Ever used anything to delay or avoid getting pregnant		
No	3547	17.7
Yes	16,440	82.3
Decision on contraceptive use		
Respondent alone/with partner	17,053	89.8
Husband/partner alone	1684	8.9
Other Interfere	250	1.3
Decision on health care visit		
Respondent alone/with partner	14,100	74.3
Husband/partner alone	4368	23.0
Other Interfere	519	2.7
Decision on visiting relative house		
Respondent alone/with partner	14,000	73.7
Husband/partner alone	3871	20.4
Other Interfere	1116	5.9
Husband Education		
No education	4011	21.1
Primary	5306	27.9
Secondary/Higher	9670	50.9
Husband or family member pressured respondent to become pregnant		
No	18,400	96.9
Yes	587	3.1
Continued study after marriage		
No	10,554	81.0
Yes	2474	19.0
Continued work after marriage		
No	847	51.0
Yes	813	49.0
Beating justified if wife goes out without telling husband		
No	18,682	93.5
Yes	1305	6.5
Beating justified if wife neglects the children		
No	18,520	92.7
Yes	1467	7.3
Beating justified if wife argues with husband		
No	18,226	91.2
Yes	1761	8.8
Beating justified if wife refuses to have sex with husband		
No	19,320	96.7
Yes	667	3.3
Beating justified if wife burns the food		
No	19,650	98.3
Yes	337	1.7
Anxiety status		
Minimal Anxiety	14,611	73.1
Mild Anxiety	4480	22.4
Moderate Anxiety	696	3.5
Severe Anxiety	200	1.0
Depression Status		
Minimal depression	14,153	70.8
Mild depression	4808	24.1
Moderate depression	762	3.8
Moderately severe depression	215	1.1
Severe depression	49	0.2

In terms of sexual and reproductive health, 65.6% of women reported their first sexual encounter before the age of 18, and 39.1% had their first child before turning 18. Approximately one-quarter (22.4%) had a history of terminated pregnancies. A majority (82.3%) used contraception to delay or avoid pregnancy. When it came to decision-making, 89.8% of women reported making choices regarding contraceptive use independently or with their partner. This trend was similarly observed in healthcare visits (74.3%) and visiting relatives (73.7%). However, a small proportion (3.1%) indicated feeling pressured by their husband or family to become pregnant.

### Justification of domestic violence

The results indicate that the majority of respondents did not justify domestic violence under various circumstances. Specifically, 93.5% believed that beating was unjustifiable if a wife went out without informing her husband, and 92.7% disagreed with it being justified if a wife neglected the children. Similarly, 91.2% stated that beating was unjustified if a wife argued with her husband. A notable 96.7% rejected the justification of beating a wife for refusing sexual intimacy, whereas 3.3% agreed it was justified. Lastly, the least justification was observed for burning food, with 98.3% opposing violence in such a scenario.

### Mental health status of the respondents

The findings based on GAD-7 score revealed that approximately three-quarters of the respondents (73.1%) experienced minimal anxiety, while a significant portion (22.4%) reporting mild symptoms. The prevalence of moderate anxiety was notably low at 3.5%, and only 1.0% of respondents reported severe anxiety. This highlights that the large proportion of women fell into the minimal or mild depression.

Based on the PHQ-9 criteria, approximately three out of four (70.8%) of the respondents experienced minimal depression, while 24.1% had mild depression. Only 3.8% reported suffering moderate depression, and 1.1% were classified as moderately severe category. Severe depression was notably impacting about 0.2% of the respondents. This indicates that the majority of women fell within the minimal to mild anxiety categories.

### Factors associated with anxiety and depression

Table 4 illustrates the results of the one-way ANOVA, examining that participant’s aged 35–49 years reported significantly higher anxiety (mean GAD score = 3.45; *p* < 0.006) and depression (mean PHQ score = 3.18; *p* < 0.001) compared to other age categories. Further, women who were widowed exhibited higher anxiety (mean GAD score = 5.06; *p*-value < 0.001) and depression (mean PHQ score = 4.98; *p* < 0.001) in contrast to those who were married. The greater level of anxiety (mean GAD score = 3.81; *p* < 0.001) and depression (mean PHQ score = 3.89; *p* = < 0.001) scores was observed among participants with no education; or those who identified as Muslim (mean GAD score = 3.19; *p* < 0.001; mean PHQ score = 3.44; *p* < 0.001). It was also found that women from low-income households were more prone to developing anxiety (mean GAD score = 3.37; *p* < 0.001) and depression (mean PHQ score = 3.53; *p* < 0.001).

**Table 4:** One way ANOVA analysis

		Depression			Anxiety		
Variables	Frequency	Mean PHQ Score	SD	Sig	Mean GAD Score	SD	Sig
Age							
15–24 Years	717	3.12	3.282	0.006	2.76	3.116	0.001
25–34 Years	6977	3.33	3.314		3.09	3.134	
35–49 Years	12,293	3.45	3.456		3.18	3.233	
Status of Marriage							
Currently married	18,987	3.32	3.333	< 0.001	3.05	3.125	< 0.001
Separated/Deserted/Divorced	457	4.60	4.152		4.51	3.878	
Widowed	543	4.98	4.373		5.06	4.047	
Education							
No education	2721	3.89	3.656	< 0.001	3.81	3.415	< 0.001
Primary	5207	3.60	3.427		3.44	3.270	
Secondary/Higher	12,059	3.19	3.314		2.85	3.075	
Household wealth status							
Poor	7502	3.53	3.458	< 0.001	3.37	3.249	< 0.001
Middle	3989	3.41	3.448		3.15	3.209	
Rich	8496	3.26	3.325		2.92	3.127	
Religion							
Islam	17,928	3.44	3.432	< 0.001	3.19	3.231	< 0.001
Hinduism/other religion	2059	2.99	3.094		2.68	2.829	
Place of Residence							
Urban	7007	3.41	3.355	0.567	3.09	3.114	0.135
Rural	12,980	3.38	3.427		3.16	3.239	
Respondent currently employed							
No	13,812	3.33	3.403	< 0.001	3.07	3.178	< 0.001
Yes	6175	3.54	3.394		3.29	3.230	
Age of having first sex							
< 18	13,112	3.45	3.410	0.001	3.22	3.212	< 0.001
≥ 18	6875	3.28	3.383		2.98	3.160	
Age of first birth							
< 18	7093	3.53	3.450	0.001	3.31	3.253	< 0.001
≥ 18	10,807	3.35	3.375		3.12	3.178	
Own mobile phone							
No	6081	3.45	3.391	0.135	3.13	3.189	0.906
Yes	13,906	3.37	3.407		3.14	3.199	
Own Smart phone							
No	7510	3.51	3.465	< 0.001	3.37	3.242	< 0.001
Yes	6396	3.20	3.329		2.86	3.124	
Ever Used Internet							
No	14,168	3.51	3.458	< 0.001	3.29	3.226	< 0.001
Yes	5819	3.11	3.242		2.76	3.088	
Ever had a terminated pregnancy							
No	15,325	3.29	3.365	< 0.001	3.01	3.127	< 0.001
Yes	4662	3.73	3.500		3.56	3.379	
Ever used anything to delay or avoid getting pregnant							
No	3547	3.65	3.738	< 0.001	3.39	3.507	< 0.001
Yes	16,440	3.34	3.322		3.08	3.122	
Decision on contraceptive use							
Respondent alone/with partner	17,053	3.30	3.339	0.065	3.03	3.142	0.134
Husband/partner alone	1684	3.44	3.247		3.16	2.930	
Other Interfere	250	3.66	3.457		3.28	3.188	
Decision on health care visit							
Respondent alone/with partner	14,100	3.37	3.395	< 0.001	3.13	3.183	< 0.001
Husband/partner alone	4368	3.22	3.154		2.89	2.929	
Other Interfere	519	2.60	2.999		2.19	2.937	
Decision on visiting relative house							
Respondent alone/with partner	14,000	3.37	3.390	< 0.001	3.14	3.191	< 0.001
Husband/partner alone	3871	3.35	3.273		2.98	2.996	
Other Interfere	1116	2.52	2.654		2.11	2.499	
Husband Education							
No education	4011	3.70	3.582	< 0.001	3.59	3.311	< 0.001
Primary	5306	3.37	3.354		3.15	3.170	
Secondary/Higher	9670	3.13	3.197		2.76	2.983	
Husband or family member pressured respondent to become pregnant							
No	18,400	3.27	3.275	< 0.001	3.00	3.075	< 0.001
Yes	587	4.83	4.561		4.47	4.155	
Continued study after marriage							
No	10,554	3.43	3.479	< 0.001	3.15	3.265	< 0.001
Yes	2474	3.01	3.049		2.58	2.803	
Continued work after marriage							
No	847	3.84	3.701	0.290	3.63	3.561	0.069
Yes	813	3.65	3.599		3.33	3.261	
Beating justified if wife goes out without telling husband							
No	18,682	3.34	3.357	< 0.001	3.08	3.154	< 0.001
Yes	1305	4.14	3.917		3.95	3.649	
Beating justified if wife neglects the children							
No	18,520	3.36	3.390	< 0.001	3.09	3.177	< 0.001
Yes	1467	3.86	3.513		3.74	3.369	
Beating justified if wife argues with husband							
No	18,226	3.36	3.410	< 0.001	3.09	3.187	< 0.001
Yes	1761	3.73	3.296		3.58	3.255	
Beating justified if wife refuses to have sex with husband							
No	19,320	3.37	3.391	< 0.001	3.11	3.175	< 0.001
Yes	667	4.01	3.650		3.95	3.669	
Beating justified if wife burns the food							
No	19,650	3.38	3.392	< 0.001	3.12	3.183	< 0.001
Yes	337	4.24	3.824		4.15	3.757	

Respondents who were currently employment indicated notably higher levels of anxiety (mean GAD = 3.29; *p* < 0.001) and depression (mean PHQ score = 3.54, p < 0.001) score compared to those who were unemployed. Participants who did not own a smartphone scored noticeably higher for anxiety (mean GAD = 3.37; *p* < 0.001) and depression (mean PHQ score = 3.51, *p* < 0.001). Similarly, individuals who had never used the internet reported significantly higher anxiety (mean GAD score = 3.29; *p* < 0.001) and depression (mean PHQ score = 3.51; *p* < 0.001) compared to internet users.

Additionally, respondents who reported having their first sexual encounter before the age of 18 (mean GAD score = 3.22, *p* < 0.001; mean PHQ score = 3.45, *p* < 0.001) or their first birth before the age of 18 (mean GAD score = 3.31, *p* < 0.001; mean PHQ score = 3.53, *p* < 0.001) exhibited significantly greater symptoms of both anxiety and depression. Women with a history of terminated pregnancy reported notably greater anxiety (mean GAD = 3.56; *p* < 0.001) and depression (mean PHQ score = 3.73; *p* < 0.001). Furthermore, respondents who had never used any contraceptive methods to delay or avoid pregnancy exhibited higher anxiety (mean GAD = 3.39; *p* < 0.001) and depression (mean PHQ score = 3.65; *p* < 0.001). Respondents whose husbands had no formal education reported significantly higher anxiety (mean GAD = 3.59; *p* < 0.001) and depressive symptoms (mean PHQ = 3.70; *p* < 0.001). Similar findings were observed among respondents whose husbands were older than 50 years (mean GAD = 3.62; *p* < 0.001; mean PHQ = 3.72; *p* < 0.001). Additionally, those who faced pressure from their husbands or family members to conceive exhibited notably higher anxiety and depression (mean GAD = 4.47; *p* < 0.001; mean PHQ = 4.83; *p* < 0.001). Higher anxiety and depressive symptoms were also reported by respondents who discontinued their studies after marriage (mean GAD = 3.15; *p* < 0.001; mean PHQ = 3.43; *p* < 0.001), made healthcare decisions independently or with their spouse (mean GAD = 3.13; *p* < 0.001; mean PHQ = 3.37; *p* < 0.001), or made decisions regarding visits to relatives'houses alone or with their partner (mean GAD = 3.14; *p* < 0.001; mean PHQ = 3.37; *p* < 0.001).

The analysis reveals that women who believed beating was justified if a wife went out without informing her husband exhibited significantly higher GAD and PHQ scores (mean GAD score = 3.95; *p* < 0.001; mean PHQ = 4.14; *p* < 0.001). Similarly, those who justified beating for neglecting children reported higher anxiety levels (mean GAD score = 3.74; *p* < 0.001; mean PHQ = 3.86; *p* < 0.001). Furthermore, respondents who justified beating if a wife argued with her husband exhibited significantly higher GAD scores (mean GAD score = 3.58; *p* < 0.001 mean PHQ = 3.73; *p* < 0.001). The justification of beating for refusing sex was similarly associated with elevated anxiety and depression (mean GAD score = 3.95; *p* < 0.001; mean PHQ = 4.01; *p* < 0.001). Finally, women who justified beating for burning food had significantly higher scores in both scales (mean GAD score = 4.15; *p* < 0.001; mean PHQ = 4.24; *p* < 0.001). These findings indicate that justifications for domestic violence are associated with higher mental distress.

### Binary logistic regression

The binary logistic regression analysis (see Table 2) indicates that participants those who were widowed (GAD: UOR = 2.23; 95% CI:1.54–2.67; *p* < 0.000; PHQ: UOR = 1.95; 95% CI:1.64–2.31; *p* < 0.000) and separated, deserted, or divorced (GAD: UOR = 1.56; 95% CI:1.21–1.95; *p* < 0.000; PHQ: UOR = 1.86; 95% CI:1.54–2.24; *p* < 0.000) had a significantly higher likelihood of developing moderate to severe anxiety and depression compared to married respondents. Women not having formal education (GAD: UOR = 1.84; 95% CI:1.69–2.02; *p* < 0.000; PHQ: UOR = 1.44; 95% CI:1.32–1.57; *p* < 0.000) and those with primary education (GAD: UOR = 1.36; 95% CI:1.19–1.56; *p* < 0.000; PHQ: UOR = 1.23; 95% CI:1.08–1.41; *p* < 0.002) were more prone to moderate to severe anxiety and depression compared to those who completed secondary or higher education.

Women from poor households (GAD: UOR = 1.35; 95% CI:1.18–1.54; *p* < 0.000; PHQ: UOR = 1.10; 95% CI:0.97–1.26; *p* = 0.005) and middle-income households (GAD: UOR = 1.17; 95% CI: 1.02–1.35; *p* = 0.027; PHQ: UOR = 1.15; 95% CI: 1.00–1.31; *p* = 0.046) were more likely to experience moderate to severe anxiety and depression compared to those from wealthy households. Furthermore, women belonging to Islam had higher odds of experiencing anxiety (GAD: UOR = 1.31; 95% CI:1.09–1.58; *p* = 0.004) compared to those from Hinduism or other religions. However, no substantial correlation was identified between religion and depression. In comparison to women with internet access, those who had never used the internet were found to have significantly greater odds of experiencing moderate to severe anxiety (GAD: UOR = 1.30; 95% CI: 1.12–1.51; *p* < 0.001) and depression (PHQ: UOR = 1.22; 95% CI: 1.06–1.41; *p* = 0.006). Women who remained employed after marriage had significantly increased chances of experiencing moderate to severe anxiety and depression (GAD: UOR = 2.00; 95% CI: 1.52–2.63; *p* < 0.000; PHQ: UOR = 1.82; 95% CI: 1.39–2.39; *p* < 0.000) compared to those who did not continue working post-marriage. We observed a significantly higher likelihood of moderate to severe anxiety (GAD: UOR = 1.14; 95% CI: 1.07–1.22; *p* < 0.000) and depression (PHQ: UOR = 1.14; 95% CI: 1.03–1.27; *p* = 0.014) among participants who were currently employed compared to those who were not employed.

The result states a significant association between a history of terminated pregnancy and anxiety and depression. Participants who had ever experienced a terminated pregnancy were found to report higher likelihood of moderate to severe anxiety (UOR = 0.72, 95% CI: 0.64–0.81, *p* < 0.000) depression (UOR = 1.31, 95% CI: 1.22–1.41, *p* < 0.000) compared to those who had never had a terminated pregnancy. This study also reported that participants who had their first sexual encounter before the age of 18 had greater chances of experiencing moderate to severe anxiety (GAD: UOR = 1.23; 95% CI: 1.15–1.31; *p* < 0.000) and depression (PHQ: UOR = 1.14; 95% CI: 1.07–1.22; *p* < 0.000) compared to those who had their first sexual encounter at or after 18. Similarly, women who had their first birth before the age of 18 were also observed to have higher odds ratio of experiencing moderate to severe anxiety and depression (UOR = 1.18; 95% CI: 1.10–1.26; *p* < 0.000) and depression (UOR = 1.14; 95% CI: 1.07–1.21; *p* < 0.000).

Women who made decisions about visiting relatives’ houses either alone or with their partners (GAD: UOR = 2.03; 95% CI: 1.44–2.86; *p* < 0.000; PHQ: UOR = 1.83; 95% CI: 1.35–2.49; *p* < 0.000); or those whose husbands or partners made these decisions (GAD: UOR = 1.89; 95% CI: 1.32–2.71; *p* < 0.000; PHQ: UOR = 1.87; 95% CI: 1.35–2.57; *p* < 0.000) showed a substantially higher chances of suffering from moderate to severe anxiety and depression.

Notably, respondents who agreed that beating was justified if the wife goes out without informing husband (GAD: UOR = 1.59; 95% CI: 1.41–1.78; *p* < 0.000; PHQ: UOR = 1.46; 95% CI: 1.30–1.64; *p* < 0.000); neglects the children (GAD: UOR = 1.45; 95% CI: 1.15–1.83; *p* = 0.002; PHQ: UOR = 1.33; 95% CI: 1.19–1.49; *p* < 0.000); wife argues with husband (GAD: UOR = 1.40; 95% CI: 1.26–1.55; *p* < 0.000; PHQ: UOR = 1.25; 95% CI: 1.12–1.38; *p* < 0.000); refuses to have sex (GAD: UOR = 1.71; 95% CI: 1.45–2.00; *p* < 0.000; PHQ: UOR = 1.49; 95% CI: 1.27–1.74; *p* < 0.000); or wife burns the food (GAD: UOR = 1.79; 95% CI: 1.44–2.23; *p* < 0.000; PHQ: UOR = 1.44; 95% CI: 1.15–1.80; *p* = 0.001) were found to have higher chances of exhibiting moderate to severe anxiety and depression than those who did not justify such actions.

### Stepwise multiple regression analysis

A stepwise multiple regression analysis has demonstrated a six-factor models (see Table 5) that can help predict both anxiety and depression among married women in Bangladesh. For Generalized Anxiety Disorder (GAD)**,** the most significant predictor is having ever experienced a terminated pregnancy, which explains 7.1% of the variance (R^2^ change = 0.071; Sig. = 0.000). The second most influential predictor is pressure from spouse or family member to become pregnant, contributing an additional 4.6% to the variance (R^2^ change = 0.046; Sig. = 0.000). Educational status ranks third, contributing 2.6% of the variance (R^2^ change = 0.026; Sig. = 0.000). The fourth factor is the justification of beating if the wife neglects the children, explaining 1.8% of the variance (R^2^ change = 0.018; Sig. = 0.012). The fifth factor is continued study after marriage, which accounts for 1.0% of the variance (R^2^ change = 0.010; Sig. = 0.039). Ultimately, the decision on visiting a relative's house contributes an additional 0.8% of the variance (R^2^ change = 0.008; Sig. = 0.035).

**Table 5:** Stepwise multiple regression analysis

Predictors (Code)	R square	R square change	F Change	Sig
Depression (PHQ-9)				
V13	.069	.069	12.739	.000
V13, V24	.110	.041	13.726	.000
V13, V24, V8	.136	.026	6.926	.009
V13, V24, V8, V20	.157	.021	4.678	.031
V13, V24, V8, V20, V26	.174	.017	4.457	.035
V13, V24, V8, V20, V26, V5	.183	.009	5.245	.022
Anxiety (GAD-7)				
V13	.071	.071	13.301	.000
V13, V20	.117	.046	12.518	.000
V13, V20, V3	.143	.026	12.699	.000
V13, V20, V3, V24	.161	.018	6.397	.012
V13, V20, V3, V24, V21	.171	.010	4.293	.039
V13, V20, V3, V24, V21, V17	.179	.008	4.464	.035

-Anxiety and depression status is the dependent variable

-V3 = Education; V5 = Religion; V8 = Age of having first sex; V13 = Ever had a terminated pregnancy; V17 = Decision on visiting relative house; V20 = Husband or family member pressured respondent to become pregnant; V21 = Continued study after marriage; V24 = Beating justified if wife neglects the children, V26 = Beating justified if wife refuses to have sex with husband,

-Sig. = Level of significance

For PHQ-based depression assessments, the most critical predictor is having ever experienced a terminated pregnancy, accounting for 6.9% of the variance (R^2^ change = 0.069; Sig. = 0.000). Justification for beating if the wife neglects children emerge as the second predictor, explaining an additional 4.1% of the variance (R^2^ change = 0.041; Sig. = 0.000). The third factor, age at first sexual experience, contributes 2.6% of the variance (R^2^ change = 0.026; Sig. = 0.009). The fourth significant predictor is pressure from the husband or family member to become pregnant, adding 2.1% of the variance (R^2^ change = 0.021; Sig. = 0.031). Justification for beating if the wife refuses sex accounts for a further 1.7% (R^2^ change = 0.017; Sig. = 0.035). Lastly, religion contributes an additional 0.9% of the variance (R^2^ change = 0.009; Sig. = 0.022).

This model highlights the interplay of reproductive, social, and cultural factors in shaping mental disorders, with the history of a terminated pregnancy emerging as the strongest predictor for anxiety and depression.

## Discussion

This study aimed to investigate the Factors Associated with Anxiety and Depression among married women in Bangladesh. Status of marriage, education, household wealth status, religion, employment status, age of first birth, ever used internet, ever had a terminated pregnancy, decision on visiting relative house, continue work after marriage are associated with higher anxiety and depression among this population. Additionally, beating justified if wife goes out without telling husband, if wife neglects the children, if wife argues with husband, if wife refuses to have sex with husband and if wife burns the food all are significantly associated with the mental health issues of Bangladeshi women.

Respondents who considered beatings justified under circumstances such as the wife goes out without telling husband, neglecting the children, argues with husband, refusing sexual relations, or burning food exhibited a greater propensity for anxiety and depression compared to those who did not condone such behavior. This aligns with global studies from Iran, Nigeria, Australia, Pakistan, and Egypt, which identify domestic violence as a critical risk factor for depression and anxiety in women [38–42]. Similarly, a study conducted in Nepal disclosed a strong correlation between emotional abuse, physical violence, and sexual assault in women and the prevalence of anxiety and depression [43]. In communities like Bangladesh, there are deeply rooted cultural norms and unwritten expectations that shape behavior, often implicitly endorsing violence against women as a socially tolerated or even routine aspect of family life [44]. Within such patriarchal contexts, women often come to accept gender-based violence as a normative part of marital relationships, interpreting abusive behavior by their husbands not as a violation but as a rightful expression of authority, believed to be in their own best interest [45]. Such normalization of domestic violence can lead to chronic psychological distress. This internalization of violence aligns with the theory of learned helplessness, which posits that when women perceive abuse as inevitable and beyond their control, they may come to accept it as reasonable, ultimately developing symptoms such as anxiety, fear, depression, and low self-esteem [46]. Global studies have identified that depression, stress-related disorders, chemical dependency, substance addiction, and suicide are repercussions associated with violence in women's life [47]. Social and economic advantages appear to confer protection against mental disorders [42]. These findings underscore the urgent need to challenge the sociocultural acceptance of domestic violence and to promote gender-equitable norms within society.

Women who made decision whether to visit relatives'house independently or with their partners, as well as those whose husbands or partners made these decisions, had significantly elevated risks of experiencing anxiety and depression. This relationship between decision-making autonomy and mental health is complex and appears to be influenced by deep-rooted socio-cultural norms. In highly patriarchal societies like Bangladesh, traditional gender roles often position men as the primary breadwinners and expect women to remain subordinate in household decision-making [48]. When women assert autonomy, such as deciding whether or not to visit relatives, it can be seen as a disruption of conventional power dynamics, potentially leading to interpersonal conflict, social backlash, or feelings of guilt and isolation [1]. Supporting this interpretation, an investigation conducted in Bangladesh indicated that women's participation in family decision-making could contest established gender norms, possibly resulting in heightened stress and deteriorating mental health. Conversely, empowered women might have a greater propensity to speak about their mental health difficulties, suggesting a potential reverse causation impact [49, 50]. In contrast, a study in India revealed that diminished autonomy correlated with an increased prevalence of common mental diseases [51]. Meanwhile, evidence from Uzbekistan revealed that women with greater decision-making power experienced more depressive symptoms, possibly due to the burden of navigating socially unaccepted roles [52, 53]. These mixed findings highlight the role of cultural context. In Bangladesh, autonomy without social support or structural change may not provide psychological relief but instead intensify emotional burdens.

Moreover, this study confirmed that married women who were currently employed and continued their work after marriage were more susceptible to experiencing anxiety and depression. This result corresponds with previous studies indicating that engaging employment may induce psychological suffering through work-to-family conflict among women [54]. While employment is often considered as a form of empowerment, it can become a source of psychological strain for women especially in contexts where they are expected to balance multiple responsibilities alongside personal and professional development [55]. In patriarchal societies like Bangladesh, women are often expected to fulfill traditional caregiving and domestic roles, even when they are engaged in full-time employment [56]. An employment position characterized by significant authority and responsibility for decision-making has been associated with an elevated risk of psychological problems [57]. A meta-analysis showed that diminished job satisfaction correlates with an increased risk of psychological discomfort, anxiety, and depression [58, 59]. As a result, employed married women often face the “double burden” of managing both professional obligations and household responsibilities, which can contribute to anxiety and depression.

Our study revealed that respondents who were widowed, separated, deserted, or divorced showed a markedly greater propensity for developing anxiety and depression in comparison to their currently married counterparts. These corresponds with prior research indicating that women in these marital settings exhibit greater emotional instability and are more prone to elevated anxiety and depression levels [60–62]. It is therefore conceivable that a correlation between marital status and mental health exists in the broader community due to evident factors such as loneliness, insufficient emotional support, and inadequate financial support in daily living [61].

Women with no education and those with only primary school exhibited a higher susceptibility to anxiety and depression than those who finished secondary or higher education. One more year of education resulted in a decreased probability of reporting symptoms associated with depression and anxiety. Individuals with higher education levels experienced less severe feelings of desperation and anxiety [16, 63]. It also reflects research on Indian women, indicating that education serves as a significant indicator of coping skills that enable women to effectively manage stress and solve problems [64]. Education elevates women's self-esteem and autonomy, potentially serving as the fundamental mechanism for the protective effects associated with higher education levels against the likelihood of mental disorders amid heightened stress levels [65, 66].

This study confirms that women from impoverished households are more vulnerable to anxiety and depression, as previous studies have established a strong correlation between poverty and mental health in women [67, 68]. Inadequate household wealth heightens daily anxieties about essential needs and restricts engagement in leisure activities, so elevating the likelihood of financial hardship, poor health, and unemployment, all of which foster the emergence of mental health problems [66, 69].

Additionally, women adhering to Islam exhibited greater likelihoods of experiencing anxiety than those from other religions. This may partly reflect the religious composition of the sample. Prior studies have linked strict religious doctrines with mental health challenges in women, often due to internalized guilt or social stigma [70, 71].

Furthermore, our study explored those participants who engaged in their initial sexual contact prior to age 18 exhibited a higher likelihood of having anxiety and depression compared to those who had their first encounter at or after age 18, which is consistent with a previous study done in Taiwan [72]. Additionally, another study also found that engaging in sexual intercourse at a young age is associated with a higher incidence of depression in adolescent girls one year later [73, 74].

Women who gave birth for the first time before the age of 18 were also shown to have an elevated chance for experiencing anxiety and depression. Past studies also demonstrated that women who had their first birth at earlier ages were more vulnerable to mental health issues [75, 76]. Moreover, evidence revealed that women who had their first child during adolescence, were more prone to mental health issues than those who were 25 years or older [77]. Various pathways may elucidate how early maternal age contributes to diminished mental health in later life. Initially, bearing a child at an early age may hinder women's education, work prospects, income, and wealth, intensifying the immediate hardships of motherhood [78] while also causing enduring adverse effects on mental health [77, 79]. Afterward, young mothers may possess diminished social support, engage in unstable or strained relationships, reside in insecure housing, and encounter heightened stigma and prejudice, resulting in adverse mental health outcomes [80–82].

Women without internet access had markedly higher probabilities of suffering anxiety and depression compared to their counterparts with internet access.

Previous studies indicate that internet use helps married women access information and social support, particularly during pregnancy and postpartum [83, 84]. Social media can also offer opportunities for leisure, foster a sense of connection, and attain professional achievement [56, 85]. Moreover, digital platforms have been shown to enhance relationship satisfaction and support women’s mental well-being [86].

Our current study revealed that women with a history of terminated pregnancies exhibit higher levels of anxiety and depression, complementing prior studies that identified a significant association between terminated pregnancies and the incidence of mental illness in women [60, 87]. Ambriz-López et al, [88]. Women who have undergone a terminated pregnancy may feel increased anxiety and depression as a result of exposure to violence, emotional turmoil, hormonal changes, societal stigma, insufficient support, and apprehensions over future fertility. These elements collectively exacerbate emotions of anxiety and depression [89, 90].

### Strengths and limitations

A key strength of this study is its ability to generalize findings across Bangladesh, as it draws on nationally representative data spanning a broad geographic region. Moreover, the application of rigorous statistical techniques, such as logistic regression and stepwise multiple regression, enhances the study’s reliability. However, certain limitations must be acknowledged. First, the cross-sectional nature of the data restricts the ability to draw causal inferences, as both predictor and outcome variables were measured simultaneously. Second, while the study includes variables related to women’s decision-making power and attitudes toward intimate partner violence (IPV), these constructs were operationalized using proxy indicators based on standard DHS questions. For instance, marital control and agency were assessed through questions on who typically makes decisions about contraceptive use, healthcare visits, and visiting relatives. Although widely used, these indicators may not fully capture the complexity of marital power dynamics, coercive control, or autonomy in practice. Additionally, some biological and behavioral factors were not clinically diagnosed, necessitating cautious interpretation of the results. The reliance on self-reported data from the two weeks preceding the survey also poses a risk of recall bias. Furthermore, certain variables linked to mental health among married women, such as drug abuse, the sudden loss of a spouse, parents, children, or close relatives, and underlying health conditions, could not be incorporated due to their absence in the dataset.

### Policy recommendations

Despite legal protections, domestic violence in Bangladesh persists due to inadequate enforcement, social stigma, and limited access to legal and mental health services [91]. Strengthening the implementation of the Domestic Violence (Prevention and Protection) Act 2010 is essential, with specialized legal and mental health support units to assist victims with professionalism and confidentiality. Mandatory gender-sensitivity training for law enforcement and service providers must be institutionalized.

Addressing mental health stigma is critical, as only 11.6% of Bangladeshi women seek mental healthcare [92]. Integrating mental health services into public healthcare, especially in marginalized areas, alongside community-based awareness programs and targeted interventions for at-risk women, can enhance support.

Economic empowerment through scholarships, vocational training, and financial literacy programs is necessary to reduce dependency and promote resilience. Digital platforms can increase access to mental health resources and peer support networks, supported by government-led awareness campaigns.

Bangladesh should adopt the “One-stop Crisis Center” model used in Nepal and other countries [93], where healthcare professionals are trained to identify and assist abuse victims comprehensively. Additionally, engaging religious and community leaders, integrating gender-sensitive curricula in schools, and introducing premarital counseling can foster cultural shifts. Engaging men and boys in anti-violence initiatives has been effective in reducing domestic violence in India [94].

## Conclusion

In terms of socioeconomic, behavioral, and biological characteristics, this study revealed a significant issue with women's mental health difficulties in Bangladesh. The core finding reveals that socio-economic and interpersonal relationship related determinants are deeply intertwined with married women’s mental health outcomes in Bangladesh. Therefore, future research should employ longitudinal designs to confirm these associations and explore additional factors, such as underlying health conditions and substance use, which were not captured in the current dataset. This study also advises local stakeholders, including policymakers, community leaders, and healthcare providers, to develop and implement culturally sensitive mental health interventions and support systems to raise awareness and serve married women with better mental health.

## Data Availability

This study used publicly available Demographic and Health Surveys Program datasets from Nepal which can be freely obtained from https://dhsprogram.com/. As a third-party user, we don’t have permission to share the data publicly in any platforms.

## Abbreviations

ANOVA: Analysis of Variance
BDHS: Bangladesh Demographic and Health Survey
DV: Domestic Violence
GAD-7: Generalized Anxiety Disorder
IPV: Intimate partner violence
MAE: Mean Absolute Error
ORs: Odds Ratios
PHQ-9: Patient Health Questionnaire
RMSE: Root Mean Squared Error
SD: Standard Deviation
Sig: Significance (*P*-value)
UOR: Unadjusted Odd Ratio
WHO: World Health Organization
